# Association between IL-2 Receptor and Severe Coronary Artery Calcification in Patients with Coronary Artery Disease

**DOI:** 10.31083/j.rcm2505186

**Published:** 2024-05-23

**Authors:** Chenyang Wang, Sheng Liu, Raimov Kamronbek, Siyao Ni, Kexin Yang, Yunxiao Yang, Daliang Zhou, Can Zhou, Chengqian Yin, Ming Zhang

**Affiliations:** ^1^Center for Coronary Heart Disease, Beijing Anzhen Hospital, Capital Medical University, 100029 Beijing, China; ^2^Department of Cardiology, First Hospital of Harbin City, 150010 Harbin, Heilongjiang, China

**Keywords:** interleukin-2 receptor, coronary artery calcification, coronary artery disease, atherosclerosis

## Abstract

**Background:**

Coronary artery calcification (CAC) is a crucial marker for coronary atherosclerosis, and the extent of CAC is closely linked to the incidence and progression of cardiovascular diseases. The interleukin-2 (IL-2) receptor (IL-2R), which plays a critical role in mediating the proliferation and differentiation of immune cells, may also be involved in the development of CAC. The study aimed to investigate the relationship between IL-2R and CAC, with the goal of providing new insights into cardiovascular diseases.

**Methods:**

In this study, we enrolled 606 patients diagnosed with coronary artery disease to assess CAC. Based on coronary artery calcification score (CACS), patients were divided into two groups: the non-severe CAC group (CACS ≤400 Agatston units, AU) and the severe CAC group (CACS >400 AU).

**Results:**

The results showed that IL-2R levels were significantly higher in patients with severe CAC compared to those with non-severe CAC (383 *vs*. 352 pg/mL, *p* = 0.002). Moreover, the level of IL-2R was positively correlated with the severity of CAC, independent of other clinical risk factors. According to Receiver Operating Characteristic (ROC) curve, the IL-2R prediction model demonstrated a good capability in distinguishing severe CAC with the Area Under the Curve (AUC) value of 0.726.

**Conclusions:**

Our study suggests that IL-2R is independently associated with the occurrence of severe CAC in coronary artery disease (CAD) patients. Additionally, IL-2R may play a crucial role in the development of advanced atherosclerosis. Consequently, therapeutic strategies targeting the IL-2/IL-2R pathway may be effective in preventing or treating CAD.

## 1. Introduction

Coronary artery calcification (CAC) is a pathological condition characterized by the abnormal accumulation of calcium and phosphorus on the inner lining of the coronary arteries. This condition is considered a clinical indicator of underlying coronary atherosclerosis [1]. Several studies have established a strong correlation between CAC severity and increases to the incidence and progression of cardiovascular diseases [2, 3, 4].

Vascular calcifications can be categorized into two types: microcalcifications and macrocalcifications. Microcalcifications manifest as small speckled or granular calcium deposits, arising from inflammation. These deposits can perpetuate further inflammation, creating a detrimental cycle of reciprocal influence [5]. In contrast, the development of macrocalcifications is associated with vascular smooth muscle cells (VSMCs). In this process VSMCs contribute to fibrosis by undergoing osteogenic transformation, resulting in the formation of uniform or patchy calcifications, commonly referred to as macrocalcifications [6, 7].

The interleukin-2 (IL-2) receptor (IL-2R) is a membrane-bound receptor expressed on T lymphocytes and plays a critical role in mediating the proliferation and differentiation of immune cells [8, 9, 10, 11]. It is involved in a variety of physiological and pathological processes, including immune responses, autoimmune diseases, and cancer [8, 9, 10, 11]. Previous studies have suggested that during the initiation and progression of atherosclerosis, the number and activity of T lymphocytes increase, events that are accompanied by a parallel increase in IL-2 expression [12, 13]. Notably, the IL-2R signaling pathway can modulate the proliferation, differentiation, and apoptosis of T lymphocytes, which may impact the occurrence and progression of atherosclerosis [14]. Nevertheless, the direct role of IL-2/IL-2R pathway in the calcification process remains unclear, highlighting the need for further research to understand its role in the underlying calcification mechanism. Moreover, emerging evidence has indicated a potential involvement of the IL-2R in the development of CAC [15]. Experimental studies utilizing human tissue samples have highlighted the presence of IL-2R expression within CAC. These observations have raised intriguing questions about the potential role of the IL-2R in the pathogenesis of CAC. Therefore, it becomes imperative to undertake an in-depth investigation to elucidate the relationship between IL-2R and CAC.

Given the potential role of IL-2R in the pathogenesis of coronary atherosclerosis, an in-depth investigation into the relationship between IL-2R and CAC is warranted. The primary objective of this study is to examine this relationship, with the aim of providing novel insights and references for further research into cardiovascular diseases. A key aspect of our investigation involves developing a predictive model to determine the likelihood of severe CAC. This model is expected to enhance our understanding of CAC’s pathogenesis and aid in identifying patients at higher risk for severe atherosclerotic complications.

## 2. Methods

### 2.1 Study Population

**Fig. 1.**
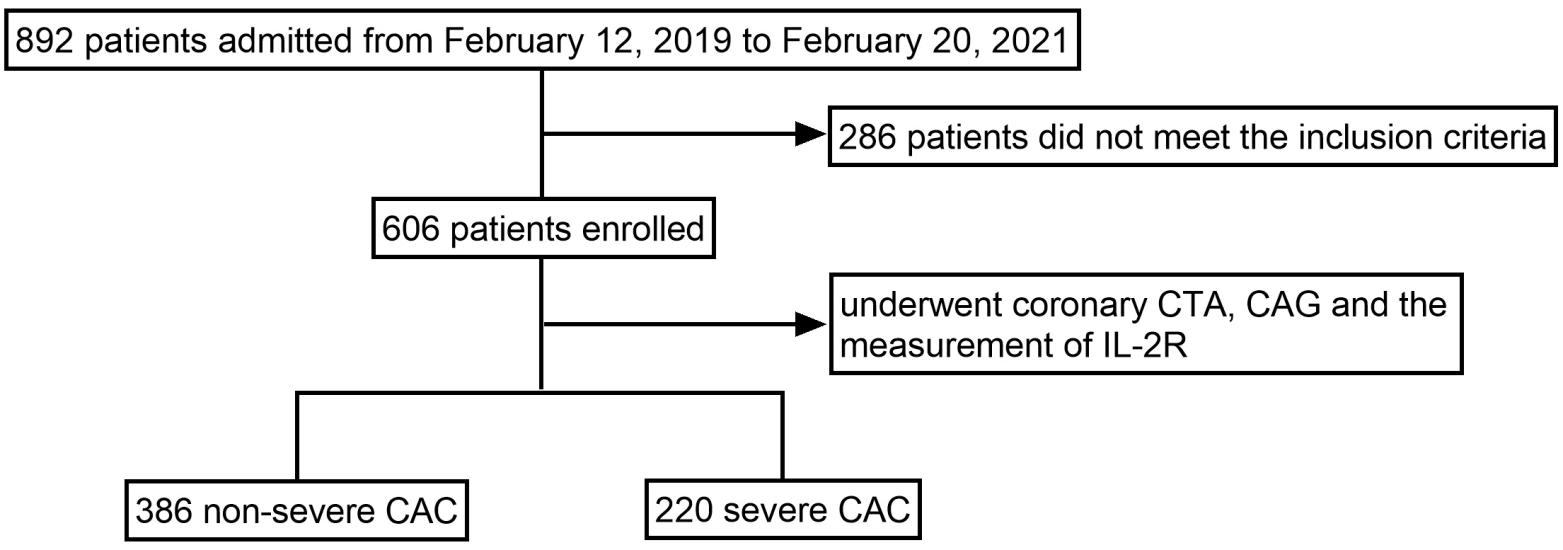
**The flow chart for enrolling and grouping**. CTA, computed tomography angiography; CAG, coronary angiography; IL-2R, interleukin-2 receptor; CAC, coronary artery calcification.

Between 12 February 2019 and 20 February 2021, we recruited 606 consecutive patients with suspected coronary artery disease (CAD) from the cardiovascular department of Beijing Anzhen Hospital. These patients underwent multi-scan computed tomography (CT) to assess coronary artery calcium. Coronary angiography (CAG) was conducted by the clinician when both the patient’s typical clinical manifestations and/or coronary computed tomography angiography (CCTA) results signify the presence of severe stenosis in at least one major coronary artery (>50%). Following the completion of CAG, all patients received a confirmed diagnosis of CAD, which was established when CAG analysis identified at least one major coronary artery with severe stenosis (>50%). Patients diagnosed with coronary artery spasm angina, valvular heart disease, active inflammatory or infectious disease, malignant tumors, severe hepatic and renal dysfunction, or autoimmune disease were excluded from the study. Additionally, patients with a history of percutaneous coronary intervention and/or coronary artery bypass grafting were also excluded. The flow chart is shown in Fig. 1.

Venous blood samples were obtained from each patient in the morning after a minimum of 12-hour fasting period, prior to CAG. Following centrifugation of the blood sample (2500 g for 10 minutes), the serum was collected and stored at a temperature of –80 °C for future assays. All procedures were conducted within a time span of 1 hour.

### 2.2 Demographic and Clinical Information

The demographic characteristics of each patient, including age, gender, body mass index (BMI), a history of hypertension, diabetes, tobacco use, and alcohol consumption, as well as the use of aspirin, clopidogrel, statins, and angiotensin-converting enzyme inhibitors (ACEI) or angiotensin receptor blockers (ARB), were recorded. The biochemical parameters, encompassing fasting blood glucose (FBG), urea, creatinine (Cr), uric acid (UA), high-sensitive C-reactive protein (hsCRP), estimated glomerular filtration rate (eGFR), glycosylated hemoglobin, type A1c (HbA1c), total triglycerides (TG), total cholesterol (TC), high-density lipoprotein cholesterol (HDL-C), and low-density lipoprotein cholesterol (LDL-C), were assessed within the Department of Biochemical Laboratory at Beijing Anzhen Hospital. The analysis was performed through a biochemical analyzer (Hitachi-7600, Hitachi, Tokyo, Japan) and a chemiluminescence immunoanalyzer (Abbott-i2000SR, Abbott, Chicago, IL, USA), employing blinded quality control specimens. The concentrations of IL-2R, were quantified through enzyme-linked immunosorbent assay (ELISA). The intra-assay and inter-assay coefficients of variation were maintained at levels below 5% and 10%, respectively.

### 2.3 Evaluation of CAC and Coronary Angiography

A dual-source CT system (SOMATOM Definition Flash, Siemens, Berlin, Germany) was employed for the performance of CCTA. All patients were examined under sinus rhythm, and breath-holding was maintained during the scan to control their heart rate below 65 beats/min. The scanning procedure encompassed the following steps: (1) acquisition of a chest location image from the trachea carina to 1 cm below the diaphragm; (2) completion of a non-enhanced CAC scan; (3) transfer of images to the workstation for calcification score computation or image post-processing, with the scanning process electrocardiogated. The scanning parameters included a tube voltage of 120 kV, tube current of 35 mA, and layer thickness of 0.6 mm. All images underwent noise reduction and artifact removal during processing. Image post-processing and analysis involved selecting phase images with minimal motion artifacts in the three different phases post-reconstruction. The Agatston method was employed for calculating the calcification score, automatically determined by the workstation computer [16]. Calcified plaques were defined as lesions with a CT value ≥130 HU (Hounsfield Units, used to quantify the density of tissues in CT imaging) and an area ≥0.5 ${\mathrm{mm}}^{2}$. Vessels of interest during image processing included the left main coronary artery (LM), left anterior descending branch (LAD), left circumflex branch (LCX), and right coronary artery (RCA). Patients were categorized into two groups based on coronary artery calcification score (CACS): the non-severe CAC group (CACS ≤400 AU [Agatston Units, a quantification of CAC]) and the severe CAC group (CACS >400 AU). The non-severe CAC group comprised patients with non-CAC (CACS = 0), mild CAC (1 ≤ CACS < 100), and moderate CAC (100 ≤ CACS ≤ 400). Subsequently, all patients underwent CAG. The severity of coronary artery stenosis was independently evaluated by two experienced interventional cardiologists, who ascertained the presence of CAD and calculated the Gensini score (GS) based on images acquired during the procedure.

### 2.4 Statistical Analysis

Categorical variables were delineated as percentages (n%), whereas continuous variables were delineated as either mean ± standard deviation for normally distributed data or as median (25th–75th percentile) for non-normally distributed data. The normality of continuous variables was assessed using the Shapiro-Wilk test. To compare quantitative variables between the non-severe CAC group and severe CAC group, either the independent student’s *t*-test or the Mann-Whitney U test was used, while the Chi-square test was used to analyze categorical variables. Spearman’s test was selected to determine the correlation between two continuous variables due to non-normal distribution of variables. Logistic regression analysis was employed to evaluate the associations among plasma biomarker levels, other measured parameters, and the level of CAC. All statistical analyses in this study were conducted utilizing IBM SPSS Statistics software version 26.0 (IBM Corporation, Armonk, NY, USA). The results attained statistical significance when the two-tailed *p*-value was below 0.05.

## 3. Results

### 3.1 Characteristics of the Study Population

Between 12 February 2019 and 20 February 2021, we enrolled a total of 606 subjects, as detailed in Table 1. This group was divided into two main cohorts based on the severity of CAC: 386 participants with non-severe CAC and 220 with severe CAC. The non-severe CAC group was further divided into three subgroups based on the coronary artery calcium score (CACS): non-CAC (CACS = 0, n = 62), mild CAC (1 ≤ CACS < 100, n = 158), and moderate CAC (100 ≤ CACS < 400, n = 166).

**Table 1.** **Clinical characteristics and IL-2R**.

Variable	Non-severe CAC (CACS ≤400)				Severe CAC (CACS >400, n = 220)	*p*
	Non-CAC (n = 62)	Mild CAC (n = 158)	Moderate CAC (n = 166)	Total (n = 386)		
Age (years)	54.60 ± 8.82	56.91 ± 9.49	59.91 ± 8.39	57.83 ± 9.12	62.98 ± 7.95	**0.031**
Male, n (%)	30 (48.4)	114 (72.2)	111 (66.9)	131 (33.9)	79 (35.9)	0.624
BMI (kg/$m^{2}$)	25.39 ± 4.01	25.78 ± 3.20	25.93 ± 3.28	25.78 ± 3.37	26.04 ± 3.22	0.636
Hypertension, n (%)	23 (37.1)	89 (56.3)	110 (66.3)	222 (57.5)	172 (78.2)	< **0.001**
Diabetes, n (%)	12 (19.4)	40 (25.3)	49 (29.5)	101 (26.2)	97 (44.1)	< **0.001**
Smoking, n (%)	4 (6.5)	11 (7.0)	18 (10.8)	33 (8.5)	32 (14.5)	**0.022**
Drinking, n (%)	2 (3.2)	8 (5.1)	15 (9.0)	25 (6.5)	17 (7.7)	0.560
Aspirin, n (%)	61 (98.4)	154 (97.5)	155 (93.4)	370 (95.9)	208 (94.5)	0.460
Clopidogrel, n (%)	41 (66.1)	99 (62.7)	96 (57.8)	236 (61.1)	154 (70.0)	**0.029**
Statin, n (%)	62 (100.0)	151 (95.6)	149 (89.8)	362 (93.8)	201 (91.4)	0.265
ACEI/ARB	14 (22.6)	46 (29.1)	60 (36.1)	120 (31.1)	73 (33.2)	0.595
FBG (mmol/L)	5.45 (5.01–6.43)	5.83 (5.12–6.43)	5.73 (5.19–6.46)	5.71 (5.12–6.43)	6.22 (5.26–7.46)	< **0.001**
Urea (mmol/L)	5.13 (4.20–5.70)	5.30 (4.50–5.90)	5.43 (4.30–6.02)	5.30 (4.40–5.90)	5.43 (4.50–6.40)	0.071
Cr (µmol/L)	66.55 (56.10–71.88)	70.35 (60.90–77.80)	70.65 (61.90–77.40)	69.90 (60.70–76.80)	71.25 (61.15–80.95)	0.174
UA (µmol/L)	344.57 (269.40–367.80)	345.74 (305.10–392.40)	345.74 (301.00–407.60)	345.74 (295.40–395.30)	345.74 (282.35–385.05)	0.624
hsCRP (pg/mL)	2.16 (0.70–3.43)	1.55 (0.59–3.26)	1.87 (0.74–2.80)	1.79 (0.68–2.89)	1.58 (0.72–2.90)	0.977
eGFR (mL/min)	99.83 (93.66–106.62)	97.29 (91.41–104.45)	93.67 (87.26–101.03)	95.90 (90.38–103.55)	93.66 (84.58–98.91)	< **0.001**
HbA1c (%)	6.00 (5.50–6.45)	5.90 (5.60–6.50)	6.00 (5.70–6.90)	6.00 (5.60–6.60)	6.30 (5.80–7.10)	< **0.001**
TG (mmol/L)	1.24 (0.87–1.55)	1.30 (1.00–1.60)	1.44 (0.96–1.94)	1.35 (0.96–1.81)	1.35 (1.03–1.77)	0.471
TC (mmol/L)	4.16 (3.40–4.51)	4.14 (3.53–4.66)	4.14 (3.51–4.68)	4.14 (3.50–4.65)	4.15 (3.57–4.67)	0.440
HDL-C (mmol/L)	1.17 (0.94–1.37)	1.15 (1.00–1.28)	1.13 (0.98–1.27)	1.16 (0.98–1.28)	1.17 (1.01–1.35)	0.181
LDL-C (mmol/L)	2.39 (1.70–2.72)	2.35 (1.80–2.88)	2.38 (1.85–2.81)	2.38 (1.80–2.87)	2.34 (1.81–2.77)	0.860
TG/HDL-C	1.17 (0.67–1.61)	1.18 (0.83–1.58)	1.32 (0.78–1.83)	1.26 (0.78–1.69)	1.21 (0.80–1.66)	0.935
GS	23.5 (20.0–34.0)	20.0 (11.0–40.0)	27.0 (16.0–48.0)	24.0 (12.0–43.0)	37.5 (19.0–70.5)	< **0.001**
IL-2R (pg/mL)	342 (271–404)	359 (290–431)	348 (308–448)	352 (292–436)	383 (318–476)	**0.002**
CACS	-	36.35 (12.00–61.70)	226.39 (158.00–304.80)	73.00 (10.00–202.00)	851.50 (552.87–1392.05)	<0.001

BMI, body mass index; ACEI/ARB, angiotensin converting enzyme inhibitors or angiotensin receptor blocker; FBG, fasting blood glucose; Cr, creatine; UA, uric acid; hsCRP, high-sensitive C-reactive protein; eGFR, effect glomerular filtration rate; HbA1c, glycosylated hemoglobin, type A1c; TG, total triglycerides; TC, total cholesterol; HDL-C, high-density lipoprotein cholesterol; LDL-C, low-density lipoprotein cholesterol; IL-2R, interleukin-2 receptor; GS, gensini score; CAC, coronary artery calcification; CACS, coronary artery calcification score. The noteworthy statistical significance in the comparison between the non-severe CAC group and the severe CAC group was indicated by the bold values.

**Fig. 2.**
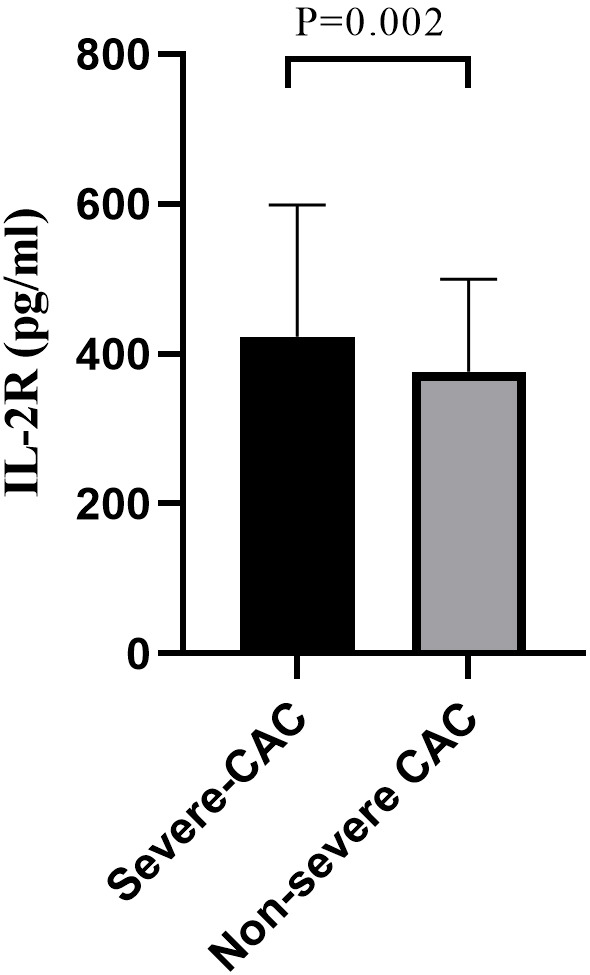
**IL-2R levels under severe CAC and non-severe CAC**. *p* values were calculated by Mann–Whitney U test. IL-2R, interleukin-2 receptor; CAC, coronary artery calcification.

Analysis of clinical characteristics revealed that the severe CAC group was older (*p* = 0.031) and had a higher prevalence of hypertension (*p* < 0.001), diabetes (*p* < 0.001), and smoking. Additionally, this group showed elevated levels of IL-2R (*p* = 0.002), fasting blood glucose (FBG, *p* < 0.001), and glycosylated hemoglobin (HbA1c, *p* < 0.001), alongside reduced estimated glomerular filtration rate (eGFR, *p* < 0.001) compared to the non-severe CAC group. Patients with severe CAC exhibited higher Gensini scores (GS), indicative of more pronounced coronary vessel stenosis, compared to patients with non-severe CAC. However, no significant differences were observed in sex, body mass index (BMI), the use of aspirin, statins, angiotensin converting enzyme inhibitors (ACEI) or angiotensin receptor blockers (ARB), total triglycerides (TG), total cholesterol (TC), high-density lipoprotein cholesterol (HDL-C), low-density lipoprotein cholesterol (LDL-C), TG/HDL-C ratio, urea, creatinine (Cr), uric acid (UA), and high-sensitive C-reactive protein (hsCRP) levels between the two groups. A box diagram illustrating interleukin-2R levels was presented in Fig. 2.

**Fig. 3.**
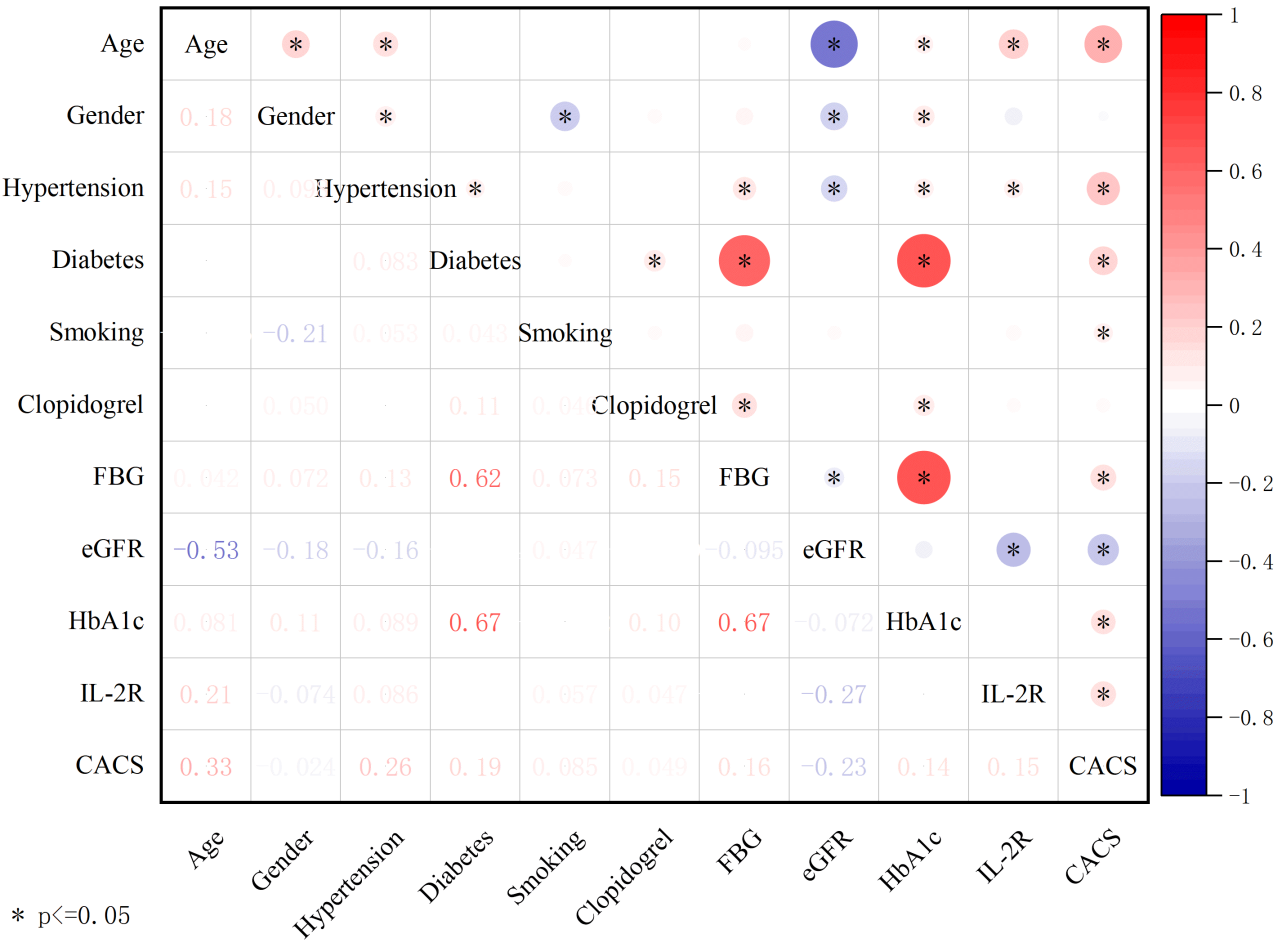
**Spearman correlation analysis was conducted on variables displaying statistically significant differences between the non-severe CAC group and the severe CAC group**. The size and color of each circle in the representation denote the magnitude of the correlation coefficient. A larger circle indicates a larger absolute value of the correlation coefficient, while the color of the circle corresponds to the value of the correlation coefficient on the color scale. Significance levels are denoted by asterisks (*p < 0.05). CAC, coronary artery calcification; FBG, fasting blood glucose; eGFR, effect glomerular filtration rate; HbA1c, glycosylated hemoglobin, type A1c; IL-2R, interleukin-2 receptor; CACS, coronary artery calcification score.

As depicted in Fig. 3, the Spearman test was employed to assess the correlation among IL-2R, CACS, and other parameters exhibiting statistically significant differences between the non-severe CAC group and the severe CAC group as presented in Table 1. The Spearman test results unveiled a negative correlation between IL-2R levels and eGFR (r = –0.275, *p* < 0.05), while positive correlations were observed with CACS (r = 0.151, *p* < 0.05), age (r = 0.207, *p* < 0.05), and hypertension (r = 0.086, *p* < 0.05). Furthermore, CACS demonstrated positive correlations with age (r = 0.333, *p* < 0.05), hypertension (r = 0.260, *p* < 0.05), diabetes (r = 0.194, *p* < 0.05), smoking (r = 0.085, *p* < 0.05), FBG (r = 0.158, *p* < 0.05), and HbA1c (r = 0.142, *p* < 0.05), and a negative correlation with eGFR (r = –0.229, *p* < 0.05).

### 3.2 Univariate and Multivariate Logistic Regression Analyses

As shown in Table 2, a univariate logistic regression analysis revealed that older age (odds ratio [OR] = 1.073, 95% confidence interval [95% CI]: 1.051–1.096, *p* < 0.001), hypertension (OR = 2.647, 95% CI: 1.813–3.864, *p* < 0.001), diabetes (OR = 2.225, 95% CI: 1.568–3.157, *p* < 0.001), smoking (OR = 1.821, 95% CI: 1.085–3.055, *p* < 0.001), clopidogrel (OR = 1.483, 95% CI: 1.041–2.112, *p* = 0.029), FBG (OR = 1.157, 95% CI: 1.068–1.253, *p* < 0.001), HbA1c (OR = 1.239, 95% CI: 1.085–1.414, *p* = 0.001), reduced eGFR (OR = 0.972, 95% CI: 0.961–0.985, *p* < 0.001), and elevated IL-2R levels (OR = 1.002, 95% CI: 1.001–1.003, *p* < 0.001) were positively associated with severe CAC (Model 1). After adjusted for age and gender, hypertension (OR = 2.438, 95% CI: 1.647–3.609, *p* < 0.001), diabetes (OR = 2.344, 95% CI: 1.624–3.383, *p* < 0.001), smoking (OR = 1.890, 95% CI: 1.087–3.287, *p* = 0.024), clopidogrel (OR = 1.464, 95% CI: 1.013–2.114, *p* = 0.042), FBG (OR = 1.167, 95% CI: 1.075–1.268, *p* < 0.001), HbA1c (OR = 1.274, 95% CI: 1.108–1.464, *p* < 0.001), and IL-2R levels (OR = 1.001, 95% CI: 1.001–1.003, *p* < 0.001) still correlated with severe CAC (Model 2). Subsequent multivariate logistic regression (age, hypertension, diabetes, smoking, clopidogrel, FBG, HbA1c, and IL-2R were taken into account) revealed that IL-2R (OR = 1.001; 95% CI, 1.000–1.002; *p* = 0.046) was an independent predictor of severe CAC (Model 3).

**Table 2.** **Univariate and multivariate analysis of factors associated with severe CAC**.

Variable	Model 1: No adjustment		Model 2: Age and sex-adjusted		Model 3: Multivariate	
	OR (95% CI)	*p*	OR (95% CI)	*p*	OR (95% CI)	*p*
Age	1.073 (1.051–1.096)	< **0.001**	-	-	1.066 (1.043–1.090)	< **0.001**
Gender	1.091 (0.771–1.543)	0.624	-	-	-	-
Hypertension	2.647 (1.813–3.864)	< **0.001**	2.438 (1.647–3.609)	< **0.001**	2.132 (1.427–3.183)	< **0.001**
Diabetes	2.225 (1.568–3.157)	< **0.001**	2.344 (1.624–3.383)	< **0.001**	1.941 (1.176–3.202)	**0.009**
Smoking	1.821 (1.085–3.055)	**0.023**	1.890 (1.087–3.287)	**0.024**	1.696 (0.961–2.991)	**0.068**
Clopidogrel	1.483 (1.041–2.112)	**0.029**	1.464 (1.013–2.114)	**0.042**	1.313 (0.892–1.934)	0.167
FBG	1.157 (1.068–1.253)	< **0.001**	1.167 (1.075–1.268)	< **0.001**	1.044 (0.930–1.172)	0.467
eGFR	0.972 (0.961–0.985)	< **0.001**	0.988 (0.974–1.002)	0.082	-	-
HbA1c	1.239 (1.085–1.414)	**0.001**	1.274 (1.108–1.464)	< **0.001**	1.003 (0.807–1.247)	0.975
IL-2R	1.002 (1.001–1.003)	< **0.001**	1.001 (1.000–1.003)	< **0.001**	1.001 (1.000–1.002)	**0.046**

OR, odds ratio; CI, confidence interval; CAC, coronary artery calcification; FBG, fasting blood glucose; eGFR, effect glomerular filtration rate; HbA1c, glycosylated hemoglobin, type A1c; IL-2R, interleukin-2 receptor. Bold values signify the statistical significance in logistics regression. Model 1: Univariate logistic regression. Model 2: Logistic regression after adjusted age and gender. Model 3: adjusted for age, hypertension, diabetes, smoking, clopidogrel, FBG and HbA1c.

### 3.3 ROC of IL-2R Prediction Model

**Fig. 4.**
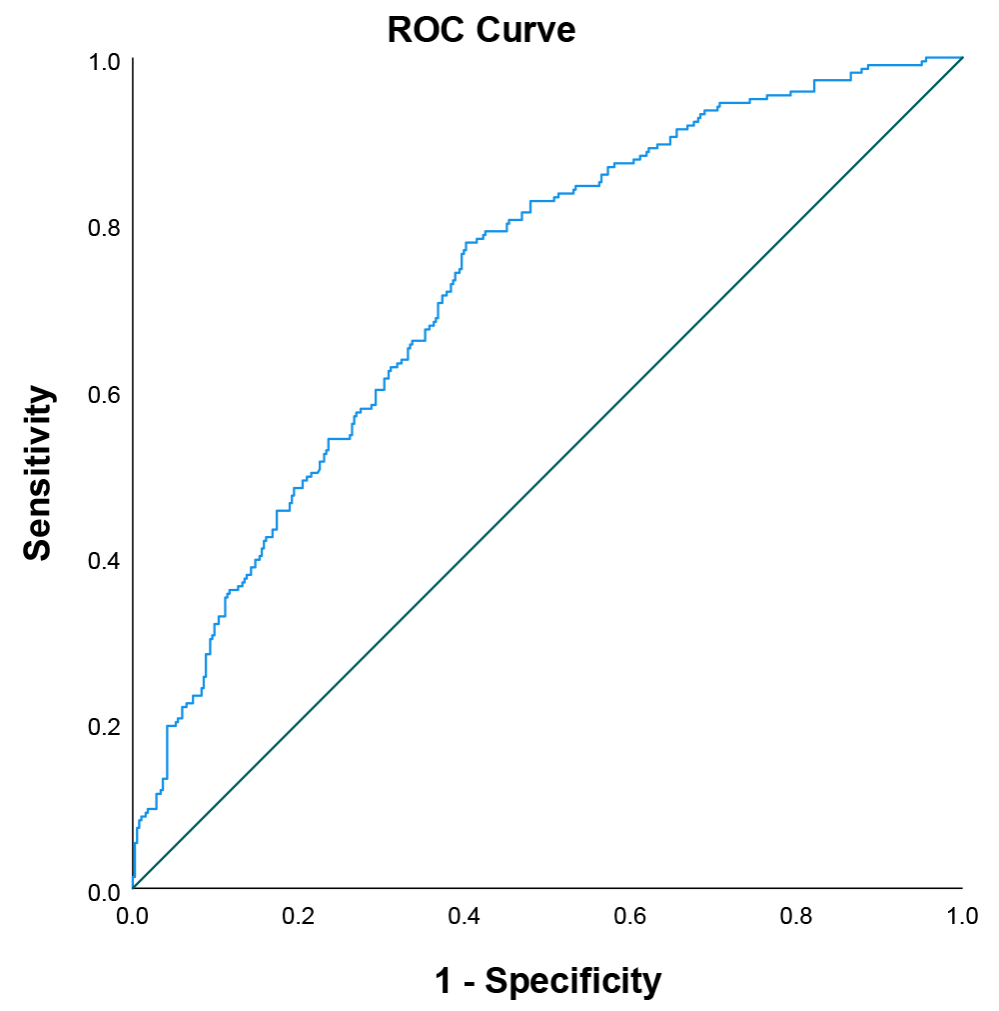
**ROC curve of IL-2R prediction model**. ROC curve, receiver operating characteristic curve; IL-2R, interleukin-2 receptor.

**Fig. 5.**
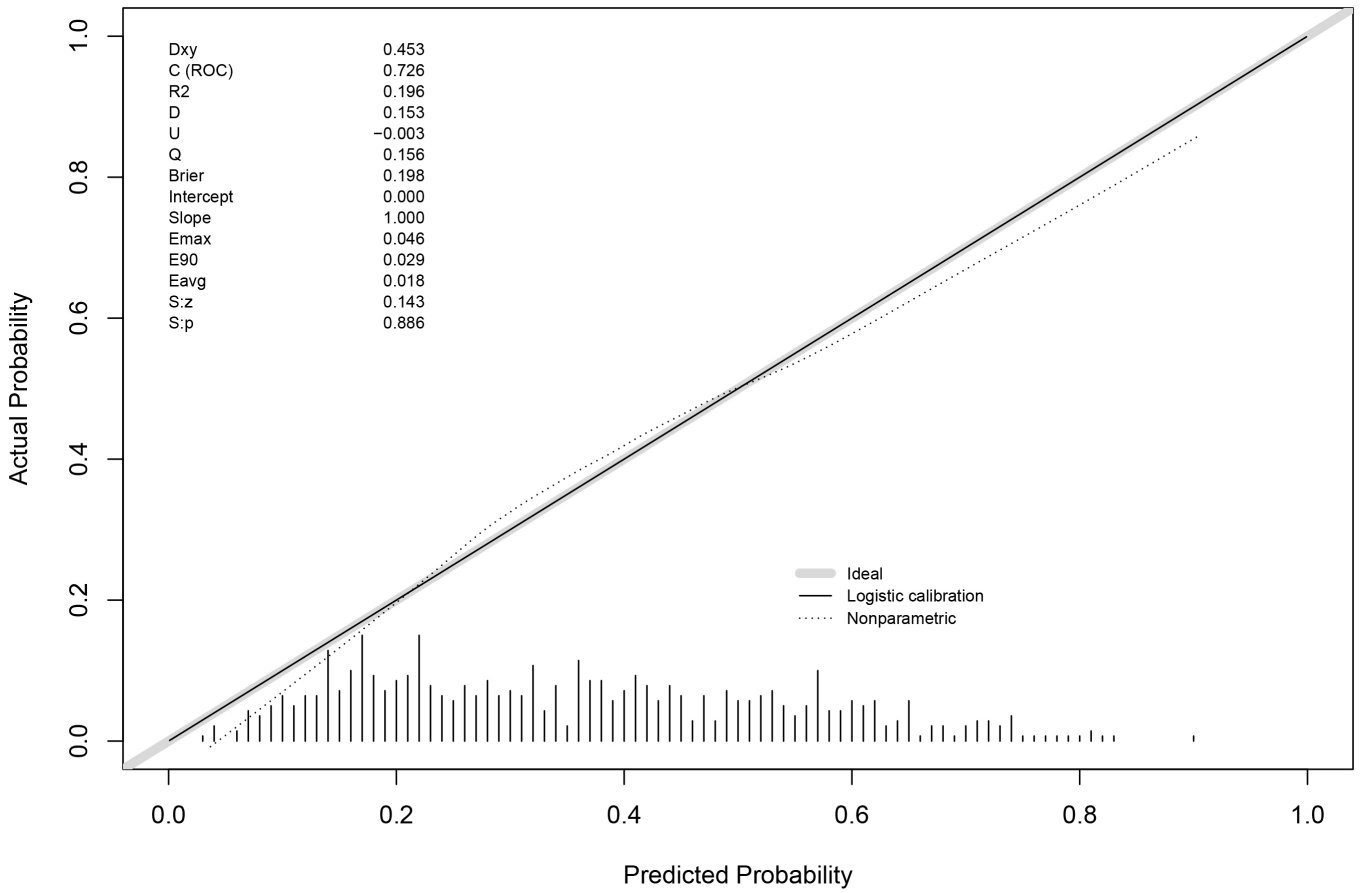
**Calibration curve of IL-2R prediction model**. IL-2R, interleukin-2 receptor.

Utilizing the outcomes derived from the multivariate logistic regression analysis, we developed a predictive model that combines age, hypertension, diabetes, smoking, and IL-2R to anticipate the presence of severe CAC. Subsequently, we generated a Receiver Operating Characteristic (ROC) curve to evaluate the model, as illustrated in Fig. 4. The model’s efficacy, indicated by the Area Under the Curve (AUC) of the ROC, was 0.726 (95% CI: 0.686–0.767), demonstrating significant predictive power (*p* < 0.001). In order to verify the accuracy of our model, we conducted the Hosmer-Lemeshow goodness-of-fit test ($X^{2}$ = 7.411, *p* = 0.594 > 0.05) and calibration curve were performed (Fig. 5).

## 4. Discussion

The current study investigated the association between IL-2R and severe CAC in a large sample of CAD patients. Our results showed that the levels of IL-2R were significantly higher in patients with severe CAC compared to those with non-severe CAC. Furthermore, IL-2R demonstrated a significant independent association with the occurrence of severe CAC, even after adjusting for potential confounding factors (OR = 1.001, 95% CI: 1.000–1.002, *p* = 0.046). We also constructed a predictive model for severe CAC based on IL-2R values, with a promising AUC of 0.726, (95% CI: 0.686–0.767, *p* < 0.001). These findings suggest that the IL-2R can effectively predict CAC in a clinical setting. Thus, IL-2R plays a crucial role in the development of severe CAC and is capable of providing novel insights into the pathogenesis of coronary atherosclerosis.

It has been established that CAC is a distinctive hallmark of atherosclerosis, a chronic inflammatory disease characterized by the accumulation of lipids, immune cells, and extracellular matrix in the arterial walls [17]. The IL-2/IL-2R pathway is an essential component of the immune response and plays a critical role in regulating the proliferation, differentiation, and apoptosis of T lymphocytes [14]. During the development of atherosclerosis, the immune system is activated, and T lymphocytes are recruited to the sites of vascular inflammation [18]. The expression of IL-2R is upregulated in activated T lymphocytes and is associated with the progression of atherosclerosis [14]. Previous studies have shown that elevated levels of IL-2R are associated with increased cardiovascular risk and adverse cardiovascular outcomes in patients with coronary artery disease. Notably, soluble IL-2R (sIL-2R) represents the soluble or circulating form of the IL-2R, which has been implicated in CAC development. Particularly, in a study investigating T lymphocyte activation in individuals with angina pectoris, it was observed that (sIL-2R) was significantly elevated in patients with unstable angina pectoris when compared to the control group (*p* <0.001) [12]. Furthermore, van der Wal *et al*. [19] observed an increase in IL-2R-positive T cells in tissue plaques from patients with unstable angina in their investigation of the immune response within coronary lesions in cases of acute coronary syndrome. Additionally, Wadwa *et al*. [15] reported higher sIL-2R levels in individuals with significantly advanced coronary calcification. This finding suggests a potential association between sIL-2R and CAC advancement. Our study further confirmed the association between IL-2R and severe CAC, independent of conventional risk factors for coronary atherosclerosis.

It has been reported that the IL-2/IL-2R pathway assumes a pivotal role in the genesis and sustenance of immune tolerance [20]. The administration of high-dose IL-2 therapy for the treatment of malignancies has been associated with the onset of autoimmune phenomena and a significant disruption of self-tolerance [21]. Mice lacking components of the IL-2/IL-2R pathway exhibit a spontaneous development of severe autoimmune disease [22]. The administration of IL-2 at low doses has been found to significantly elevate Treg cell levels, restore immune tolerance, and effectively curtail plaque development and inflammation in atherogenic-prone mice [23, 24]. The ongoing LILACS and IVORY clinical trials, conducted by Zhao *et al*. [25] and Sriranjan *et al*. [26], advocate the expansion of Tregs using low-dose IL-2 for the treatment of stable ischemic heart disease and acute coronary syndrome. The mechanism underlying the association between the path of IL-2/IL-2R and CAC is not fully understood. It is hypothesized that IL-2R may contribute to the development of atherosclerosis by promoting T lymphocyte activation and proliferation. Additionally, IL-2R may also play a role in regulating the differentiation and activation of regulatory T cells, which are important for maintaining immune tolerance and suppressing excessive immune responses [20]. Further studies are needed to elucidate the mechanisms underlying the association between IL-2R and CAC severity.

Given the well-documented link between chronic kidney disease and CAC [27, 28], our model incorporated eGFR as a variable. To investigate whether adjusting for eGFR would affect the significance of IL-2R, we included eGFR in our analysis. However, we found that the inclusion of eGFR did not have any impact on the significance of IL-2R in the regression model. Moreover, eGFR itself did not emerge as a significant predictor of CAC progression.

There are some limitations to our study. First, the study was cross-sectional, and we cannot establish causality between IL-2R and CAC severity. Second, the focus was exclusively on IL-2R measurements. We did not assess other inflammatory markers which may also play a role in the pathogenesis of atherosclerosis. Third, our study was conducted on a Chinese CAD patient cohort, thus our findings may not be generalizable to different ethnic or demographic groups.

## 5. Conclusions

In conclusion, our study suggests that IL-2R is independently associated with the occurrence of severe CAC in CAD patients. This finding suggests that IL-2R may play a crucial role in the development of advanced atherosclerosis. Consequently, targeting the IL-2/IL-2R pathway is a promising therapeutic strategy for preventing or treating CAD.

## Data Availability

The datasets presented in this article are not readily available because of the protection of personal data and privacy restrictions. Requests to access the datasets should be directed to MZ, zhangming2279@hotmail.com.
